# Successful xenotransplantation of testicular cells following fractionated chemotherapy of recipient birds

**DOI:** 10.1038/s41598-023-45019-0

**Published:** 2024-02-07

**Authors:** Marcel Henrique Blank, Allison Jun Taguchi Kawaoku, Bruno Rogério Rui, Ana Claudia Oliveira Carreira, Thais Rose dos Santos Hamilton, Marcelo Demarchi Goissis, Ricardo José Garcia Pereira

**Affiliations:** 1 Department of Animal Reproduction, College of Veterinary Medicine and Animal Science, University of São Paulo, Av. Duque de Caxias Norte 255, Pirassununga, SP CEP 13635-900 Brazil; 2 Hendrix Genetics Brazil, Estrada Municipal Slt-161 Km 08, Salto, 13328-400 Brazil; 3 Cell and Molecular Therapy Center (NUCEL), Medical School, University of Sao Paulo, Rua Pangaré 100, São Paulo, 05360-130 Brazil

**Keywords:** Biotechnology, Stem cells, Adult stem cells, Zoology

## Abstract

An essential step in the success of germ cell transplantation is the preparation of the recipient’s testicular environment to increase the availability of stem cell niches. However, most methods for this purpose in birds face serious limitations such as partial germ cell depletion, high toxicity and mortality, or the need to use expensive technologies. Here, we validated a simple and practical technique of transferring quail testicular cells into chicken testes depleted of endogenous spermatozoa by fractioned chemotherapy (20 mg/kg/week busulfan for 5 weeks). This protocol resulted in a very low mortality of the treated day-old chicks and, despite maintenance of androgenic activity, sperm production was decreased by 84.3% at 25 weeks of age. NANOG immunostaining revealed that very few to no germ cells were present following treatment with 20 and 40 mg/kg, respectively. RT-qPCR data also showed that *c-MYC* and *NANOG* expression declined in these treatments, but *GRFα1* and *BID* expressions remained unaltered among groups. After xenotransplantation, quail germ cells were immunodetected in chicken testes using a species-specific antibody (QCPN), and quail ovalbumin DNA was found in seminal samples collected from chicken recipients. Together, these data confirm that fractionated administration of busulfan in hatchlings is a practical, effective, and safe protocol to prepare recipient male birds capable of supporting xenogeneic spermatogenesis.

## Introduction

The management of genetic diversity in birds is challenging because of the constraints in preserving and handling avian gametes. The enormous amount of yolk in megalecithal eggs makes oocyte and embryo freezing unfeasible, and the oviparous reproductive pattern of birds limits the use of nuclear transfer^1–3^. In addition, avian spermatozoa are greatly vulnerable to freezing due to the high amounts of polyunsaturated fatty acids (PUFAs), low protein levels and low cholesterol/phospholipid ratio in their cell membranes^4,5^, circumstances that together make post-thaw fertility extremely low^3^. Worse still, the lack of reproductive selection and the reduced genetic variability in endangered birds lead to very low fertility and hatchability rates hindering the application of other reproductive biotechnologies^6^.

Alternatively, avian primordial germ cells (PGCs) and spermatogonial stem cells (SSCs) have already been successfully transplanted and cryopreserved^7,8^, opening up new possibilities for the development of germplasm banks. Unlike spermatozoa or oocytes, these cells can be propagated in vitro and then frozen or transferred to either recipient embryos or adult birds^9,10^. The efficiency of germ cell transplantation (GCT) can be optimized in two ways: (1) by increasing the number and purity of donor stem cells to be injected; and (2) by injecting the donor cells into recipient testes with little or no endogenous spermatogonia^11^. In the latter case, researchers have recently succeeded in producing genetically engineered sterile chickens as recipients for GCT^8,12,13^. However, genetic ablation of the recipients’ germ cells demands high investments in laboratory infrastructure, specialized workforce, and regulations for the production, handling, movement, confinement and disposal of genetically modified organisms (GMOs). For these reasons, the development of simple and low-cost protocols for the preparation of recipient birds may constitute an interesting alternative for facilities with limited human and financial resources and for those who want to avoid the entire legal framework linked to GMO production.

To this end, two approaches have been currently employed in avian species: (1) busulfan injection into recipient embryos and (2) fractionated testicular irradiation^1,14–19^. Injections of busulfan into egg yolks have long been exploited in the preparation of partially sterile embryos, but post-treatment compensatory proliferation of endogenous PGCs restricts its use in SSC transplantation into young or adult recipients^20^. In parallel, local irradiation of the testes generates few systemic effects and preserves testicular somatic cells, yet it requires sophisticated and expensive equipment making it impractical in many situations^1,19^. Hence, in order to facilitate germ cell depletion prior to SSC transfers, researchers investigated the administration of busulfan in immature and adult roosters^21–23^. Nevertheless, the tolerance of these birds to chemotherapeutic treatment is apparently low as acute toxicity and death were observed at doses ranging from 35 to 60 mg/kg^22,23^. To mitigate such impact, Tagirov and Golovan^23^ examined the application of fractionated doses of busulfan (40 + 20 mg/kg–10 days apart) in pubertal-age roosters, which successfully suppressed spermatogenesis without severe complications to the organism. These authors also reported that fractionated doses (20 + 20 mg/kg) produced more efficient sterilization than single injections (40 mg/kg) with reduction of 23% in sperm concentration and delayed spermatogenesis by 8 days when compared to single-dose subjects.

Although promising, refinements and further studies are still needed to optimize these fractionated chemotherapy regimens in recipient birds, particularly at younger ages (e.g. one-day-old chicks). This is because the use of neonates as recipients markedly increased the number and the length of donor cell colonies after SSC transplantation due to (1) the greater accessibility to SSC niches (as a result of the single layer of germ cells lining the seminiferous tubules and the absence of Sertoli cell junctions), and (2) the greater niche support to SSC expansion (through hormonal and growth factors)^11,22–27^. Besides, another advantage of using newly hatched chicks during busulfan treatment relies on the previous knowledge that children and young animals demonstrate a greater tolerance to chemotherapy when compared to adults^28,29^. Therefore, this study was designed to: (1) assess survival, tolerability, testosterone secretion, sperm production, and depletion of endogenous germ cells in chicken recipients subjected to fractionated doses of busulfan at a young age, and (2) determine whether or not this approach can be successfully applied to transfer cryopreserved germ cells from another species. From our findings, we believe this to be a simple, inexpensive and effective strategy for SSC transplantation in a variety of bird species in which recipient production by genetic ablation may be more complex and time-consuming. Nevertheless, advances in both the selection and in vitro propagation of SSCs should increase the efficiency of these processes given the small number of SSCs recovered in mature testes.

## Results

### Fractionated chemotherapy (FC) on chick health and survival

**Figure 1:**
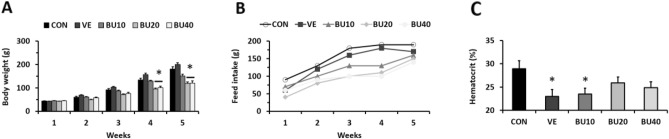
Weekly progression of (**A**) body weight and (**B**) feed intake of one-day old chicks during treatment with fractionated doses of busulfan. (**C**) Effects of busulfan treatment on hematocrit of 6-week old chicks at the end of the chemotherapy regimens. Asterisks indicate statistically significant differences from control group (CON – P < 0.05). Vehicle injection (VE – DMF + distilled water), busulfan injections at concentrations of 10, 20 and 40 mg/kg (BU10, BU20, and BU40 groups, respectively).

All deaths recorded in our study (9 out of 100 birds) occurred regardless of treatment within 7 days from the first injection, possibly due to incorrect application of the solutions into the gut that resulted in intestinal necrosis (confirmed at necropsy). Although we noted that body weights and feed intake in treated groups with 20 mg/kg and 40 mg/kg (BU20 and BU40, respectively) were lower than control (CON) (P < 0.05; Fig. 1A and B), the hematocrit tests revealed that just vehicle (VE) and treated group with 10 mg/kg (BU10) exhibited considerably decrease their red blood cell concentrations after the end of treatment (Fig. 1C).

### FC on testosterone levels and sperm parameters

**Figure 2:**
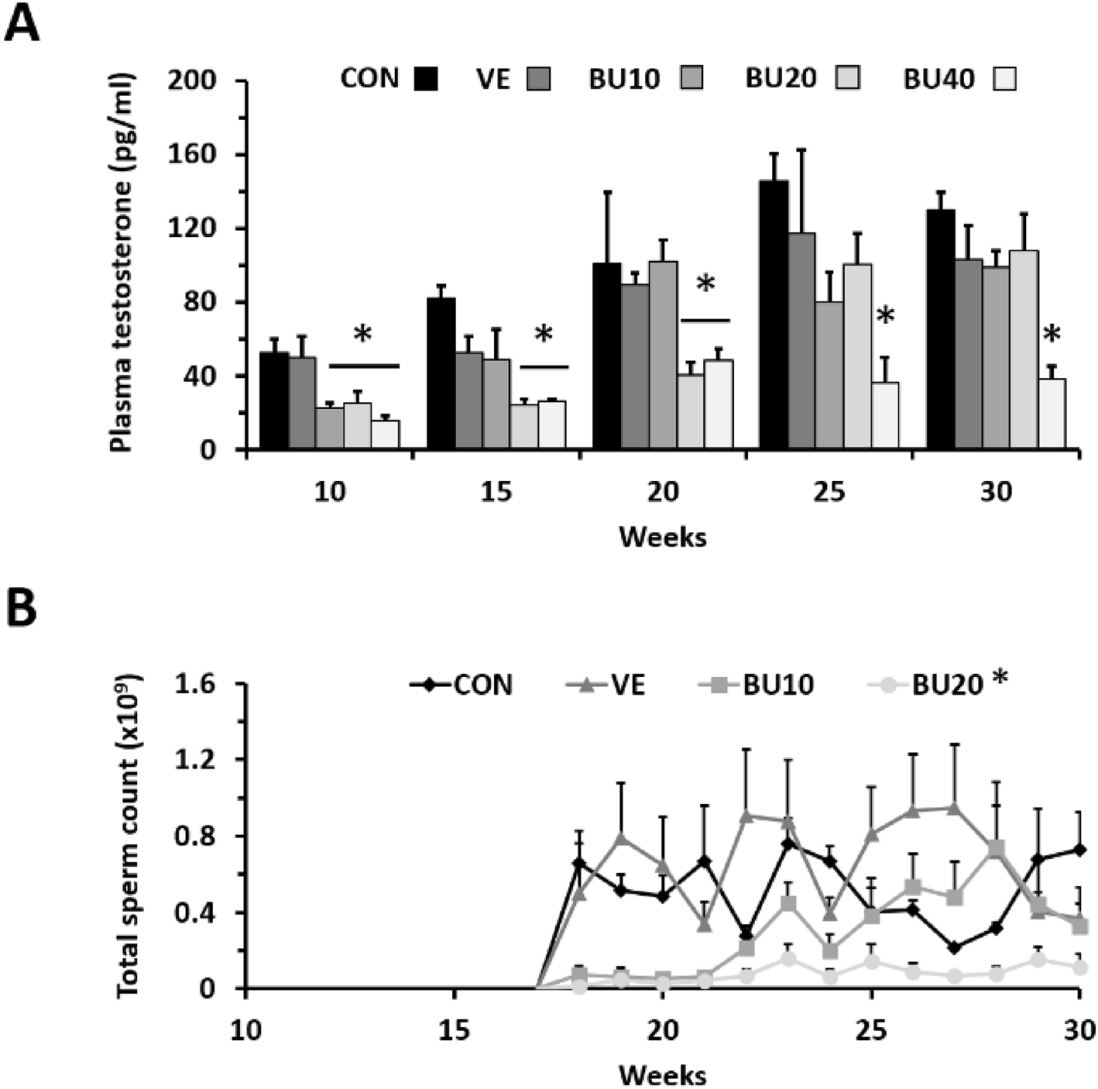
(**A**) Plasma testosterone levels and (**B**) sperm production of chicken roosters treated with different fractionated doses of busulfan during their first five weeks of life. Data are reported as mean ± SEM. Roosters subjected to the highest busulfan dose (BU40) failed to produce sperm from 18 to 30 weeks of age. Asterisks indicate statistically significant differences from control group (CON – P < 0.05). Vehicle injection (VE – DMF + distilled water), busulfan injections at concentrations of 10, 20 and 40 mg/kg (BU10, BU20, and BU40 groups, respectively).

At 10 weeks of age, testosterone levels in all treated cockerels were lower than CON and VE regardless of busulfan dose (P < 0.05 – Fig. 2A). Afterwards, individuals from BU10 and BU20 apparently restored their androgenic activity at 15 and 25 weeks of age, respectively, whereas BU40 remained at low levels throughout this study. In parallel, sperm production in BU10 recovered to CON and VE levels at 23 weeks of age; while BU20 produced marginal sperm counts over the monitoring period (0.01–0.15 × 10^9^ spermatozoa per ejaculate – Fig. 2B). Roosters subjected to the highest busulfan dose (BU40) failed to produce sperm from 18 to 30 weeks of age. Motility parameters of spermatozoa produced by busulfan-treated roosters were similar to those observed in controls and VE.

### FC on gonadosomatic index (GSI) and testicular parenchyma

**Figure 3:**
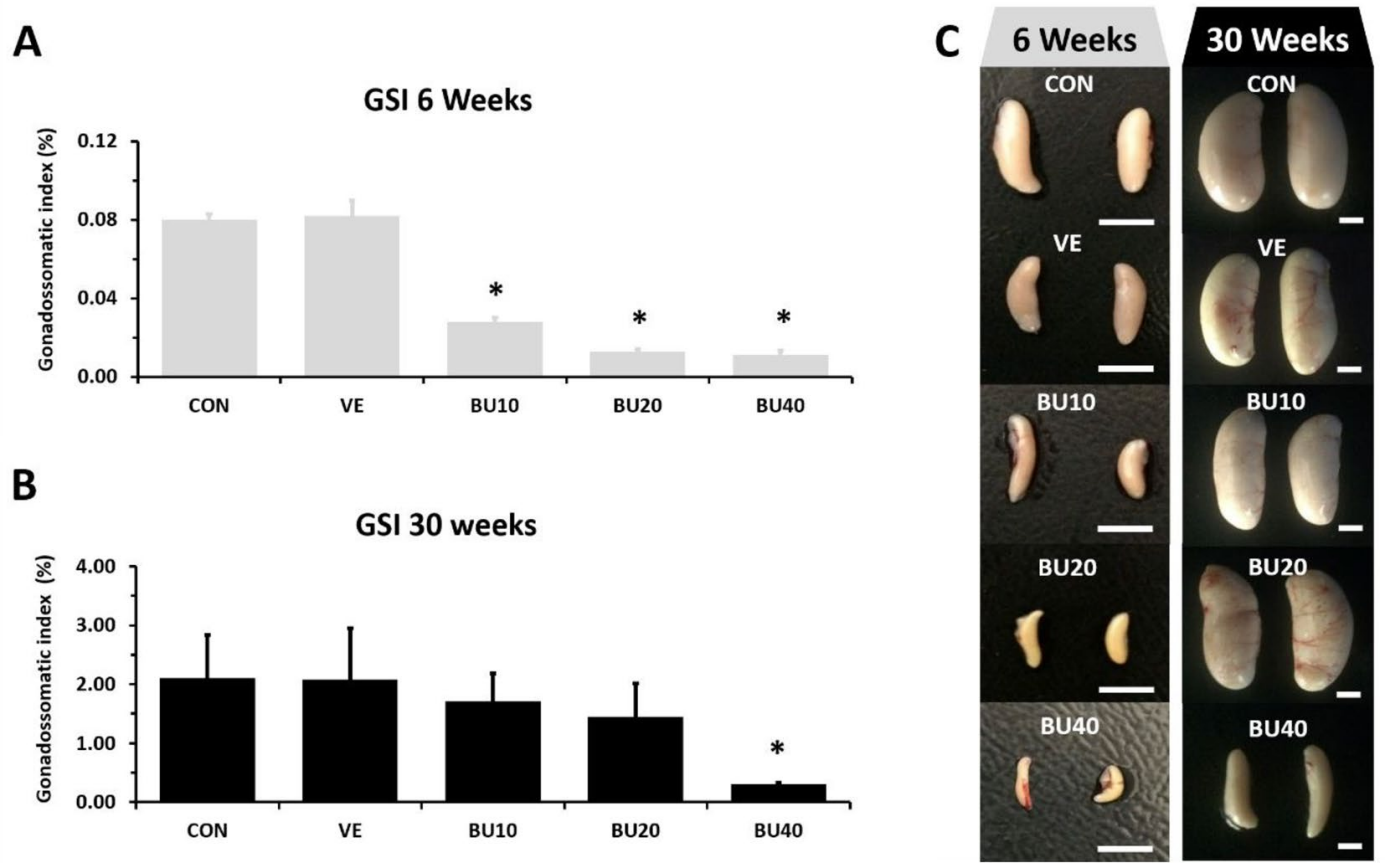
Gonadosomatic index (GSI) of chicken roosters at (**A**) 6 and (**B**) 30 weeks of age after treatment with different fractionated doses of busulfan during their first five weeks of life. (**C**) Macroscopic comparison of chicken testes from control (CON), vehicle (VE – DMF + distilled water), and busulfan groups (BU10, BU20, and BU40) at 6 and 30 weeks of age. Asterisks indicate statistically significant differences from control group (CON – P < 0.05). Scale bars represent 1 cm.

**Figure 4:**
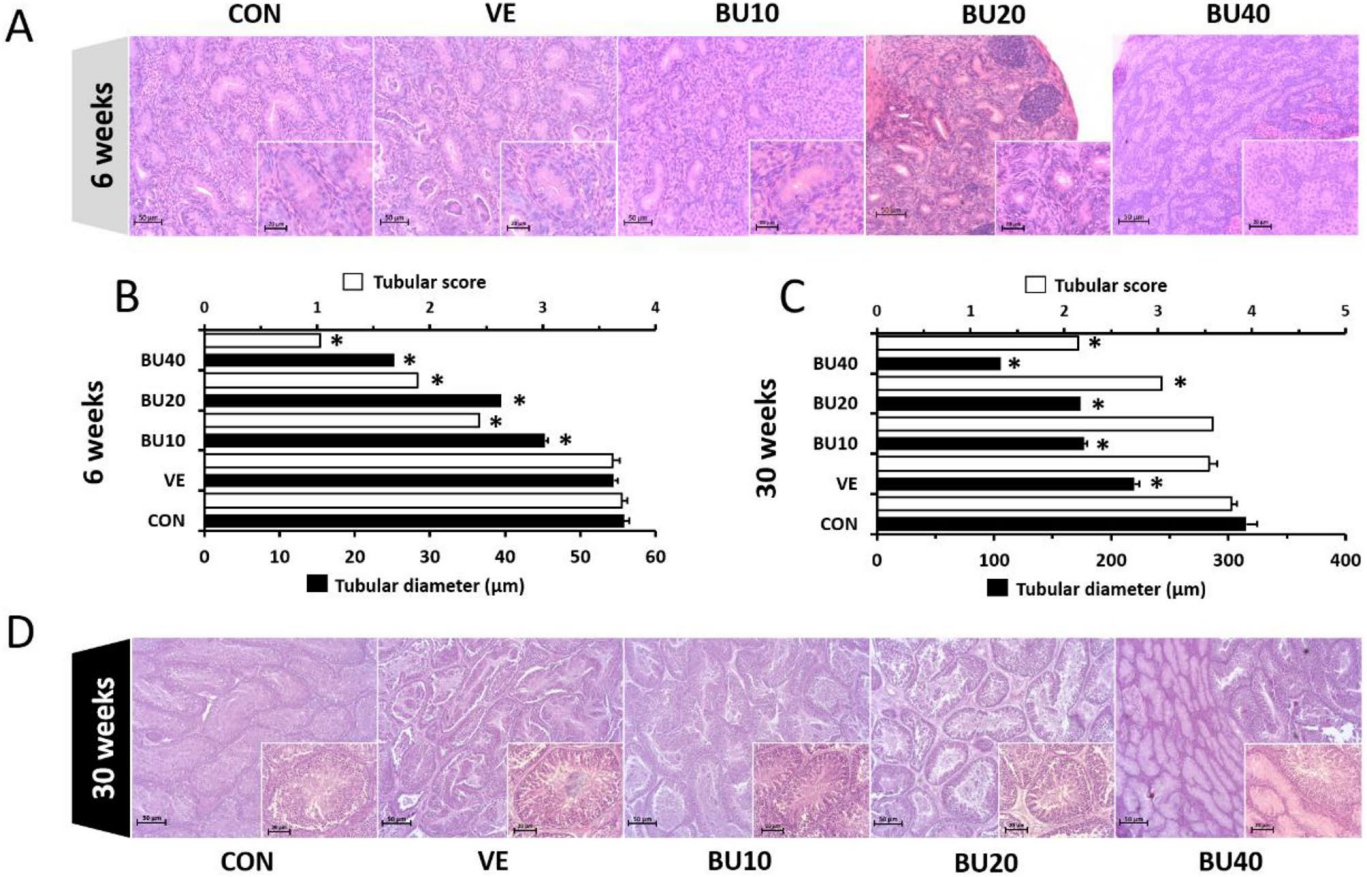
Histological analysis of the testicular parenchyma in control and busulfan-treated chicks. (**A**) Light micrographs of testis sections from 6-week old chicks in control (CON), vehicle (VE), and busulfan-treated groups (BU10, BU20 and BU40 correspond to doses of 10, 20 and 40 mg/kg) (H&E 200X; Insets 400X). (**B**) and (**C**) Mean (± SEM) seminiferous tubular diameter and score in experimental groups at 6 and 30 weeks of age, respectively. (**D**) Light micrographs of testis sections from 30-week old roosters in control, vehicle, and busulfan-treated groups (H&E 200X; Insets 400X). Asterisks indicate statistically significant differences from control group (CON – P < 0.05). Scale bars represent 50 μm (20 μm in insets).

Increasing doses of busulfan led to gradual declines in GSI of 6-week-old chicks implying suppression of endogenous spermatogenesis (P < 0.05 – Fig. 3A and C). However, busulfan effects on testes weight did not appear to persist to 30 weeks of age, except for BU40, which demonstrated a seven-fold decrease in GSI when compared to CON and VE (P < 0.05 – Fig. 3B and C). BU20 at later ages resulted in a wide range of gonadal masses varying from 0.23 to 2.29%. Histology of the testes confirmed a dose-dependent reduction in the diameter of the seminiferous tubules at 6 weeks of age (P < 0.05), with changes in the epithelium that ranged from slight numerical decreases of germ cells in tubule cross sections (BU10) to extensive loss of cells inside tubules (mostly observed in BU40) (Fig. 4A). These disturbances were also detected by our rating system, since BU10, BU20 and BU40 groups displayed lower average tubular scores than CON and VE groups (P < 0.05) (Fig. 4B). At 30 weeks of age, decreases in tubular diameter were still noticeable at all busulfan doses, although significant changes in tubular score were only perceived in roosters treated with 20 and 40 mg/kg (BU20 and BU40 – Fig. 4C and D).

**Figure 5:**
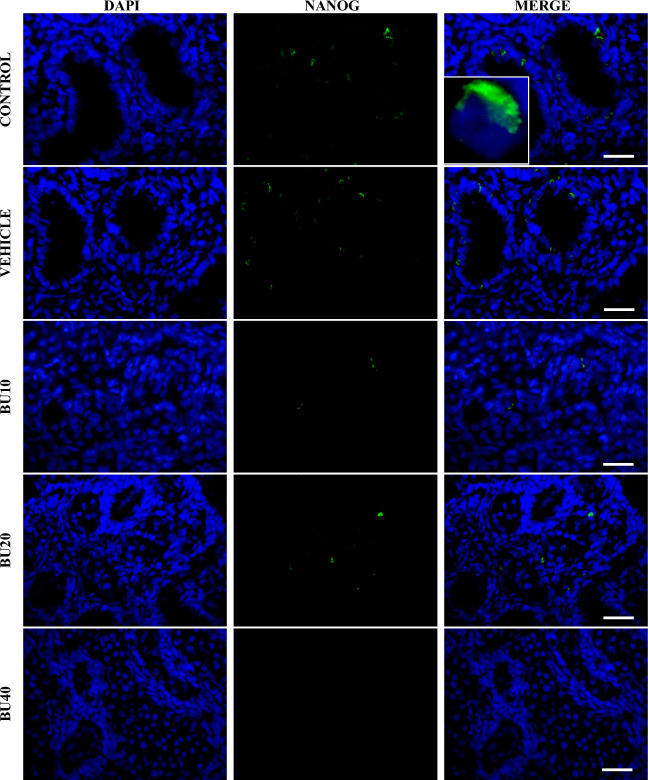
Immunofluorescent-stained cryosections of testicular parenchyma from control and busulfan-treated chicks using anti-Nanog antiserum. Testis sections of 6-week old chicks from control and vehicle groups (CON and VE, respectively) showing cytoplasmic expression of NANOG in many germ cells. Marked reduction of germ cells expressing NANOG in testis from chicks treated with busulfan at 10 mg/kg (BU10). Very few cells expressed NANOG in testis sections from chicks treated with busulfan at 20 mg/kg (BU20). No expression of NANOG was detected in chicks treated with busulfan at 40 mg/kg (BU40). Each scale bar indicates 50 μm.

NANOG protein, a marker of pluripotent and germ cells, was detected in the nuclei of some cells in stage EGK-X blastoderms and primordial gonads collected from embryos in stage 28 HH (used as positive controls – Supplementary Fig. S1). Immunofluorescence of adult testes revealed that many germ cells in the seminiferous tubules of CON and VE chicks expressed NANOG in their cytoplasm. The number of testicular cells expressing NANOG dropped sharply in chicks treated with busulfan at 10 mg/kg (BU10), while very few cells stained for NANOG in sections from BU20 chicks. NANOG expression was not found in any of the testis sections from BU40 group (Fig. 5).

### FC on the expression of apoptotic, pluripotency and germ cell markers

**Figure 6:**
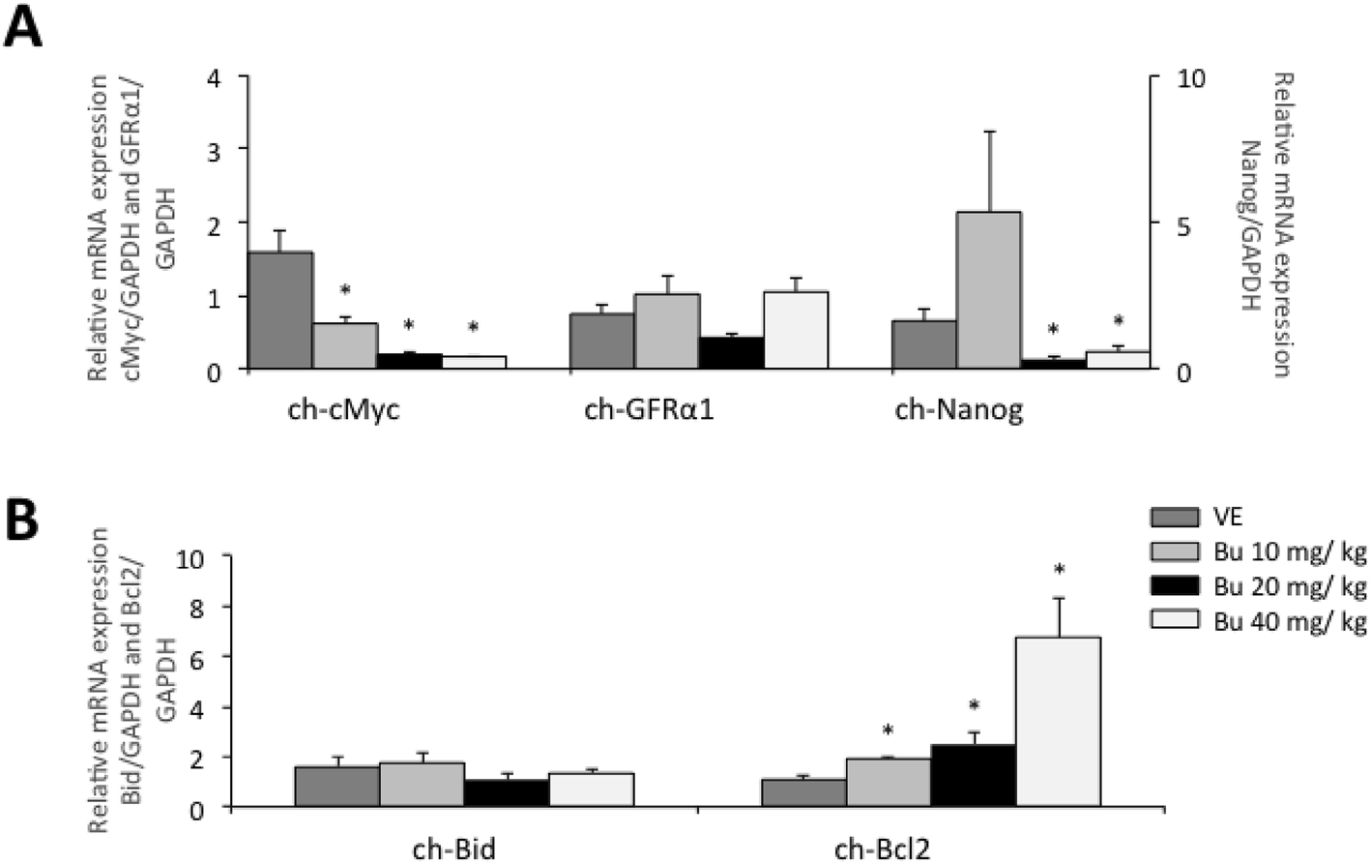
Relative mRNA expression of genes for pluripotency (*c-Myc*), primordial germ cells (*Nanog*), spermatogonial stem cells (*GFRα1*), apoptosis (*Bid*) and anti-apoptosis (*Bcl-2*) in testes from 6-week old chicks treated with fractionated doses of busulfan. (**A**) Values are expressed as arbitrary units of *c-Myc*, *GFRα1* and *Nanog* mRNA normalized against the expression levels of GAPDH amplified from the same template, relative to the expression observed in controls. (**B**) Values are expressed as arbitrary units of *Bid* and *Bcl-2* mRNA normalized against the expression levels of GAPDH amplified from the same template, relative to the expression observed in controls. Asterisks indicate statistically significant differences from control group (CON – P < 0.05). Vehicle injection (VE – DMF + distilled water), busulfan injections at concentrations of 10, 20 and 40 mg/kg (BU10, BU20, and BU40 groups, respectively).

RT-qPCR was performed in testes at the end of treatment to estimate the effectiveness of fractionated doses of busulfan in the reduction of endogenous germ cells (Fig. 6). All busulfan groups expressed lower c-Myc levels compared to VE group (P < 0.05), although expression of this gene in BU10 was considerably higher than in BU20 and BU40 groups (P < 0.05). There were no differences between groups regarding GFRα1 expression levels (P > 0.05) whereas Nanog was expressed at much lower level in BU20 and BU40 (5- and threefold less, respectively, P < 0.05). Expression levels of Bid were approximately equal for all groups (P > 0.05). Dose-dependent increases in Bcl2 expression levels were detected with BU10 and BU20 groups exhibiting intermediate rises (1.7- and 2.3-fold, respectively) compared to VE, and BU40 markedly higher expression levels (6.1-fold) (P < 0.05).

### Detection of quail germ cells in chicken recipients prepared by FC

**Figure 7:**
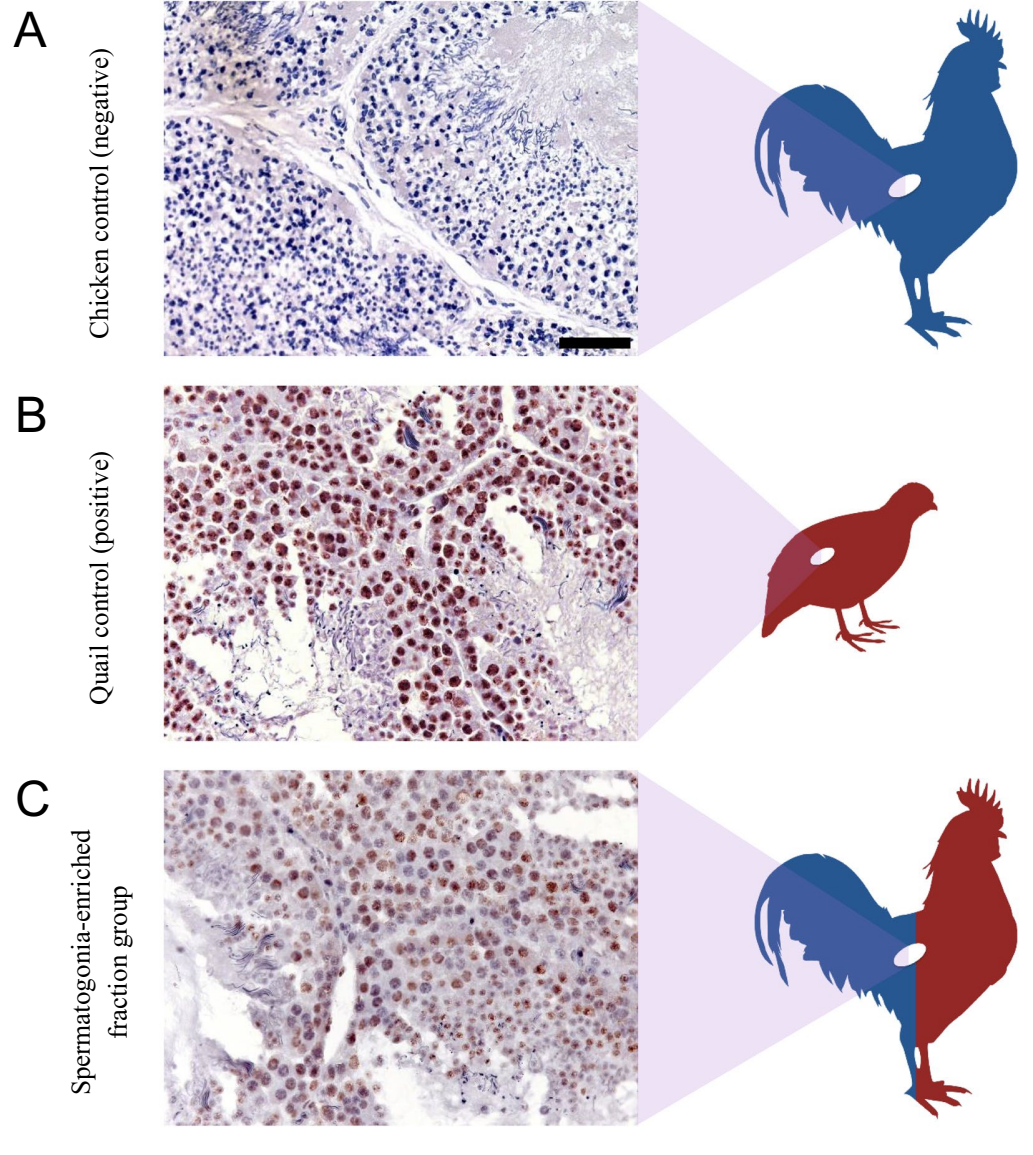
Immunodetection of quail germ cells using quail specific antibody (QCPN, a nuclear marker) in testes of transplanted chicken recipients. (**A**) Seminiferous tubules of adult chicken without interspecific transplantation and, consequently, without labeling for quail cells (negative control). (**B**) Seminiferous tubules of adult quail showing nuclear labelling of germ cells with QCPN (positive control). (**C**) Seminiferous tubules of adult chicken recipient transplanted with spermatogonia-enriched cell fraction from quail and exhibiting nuclear labeling of germ cells.

**Figure 8:**
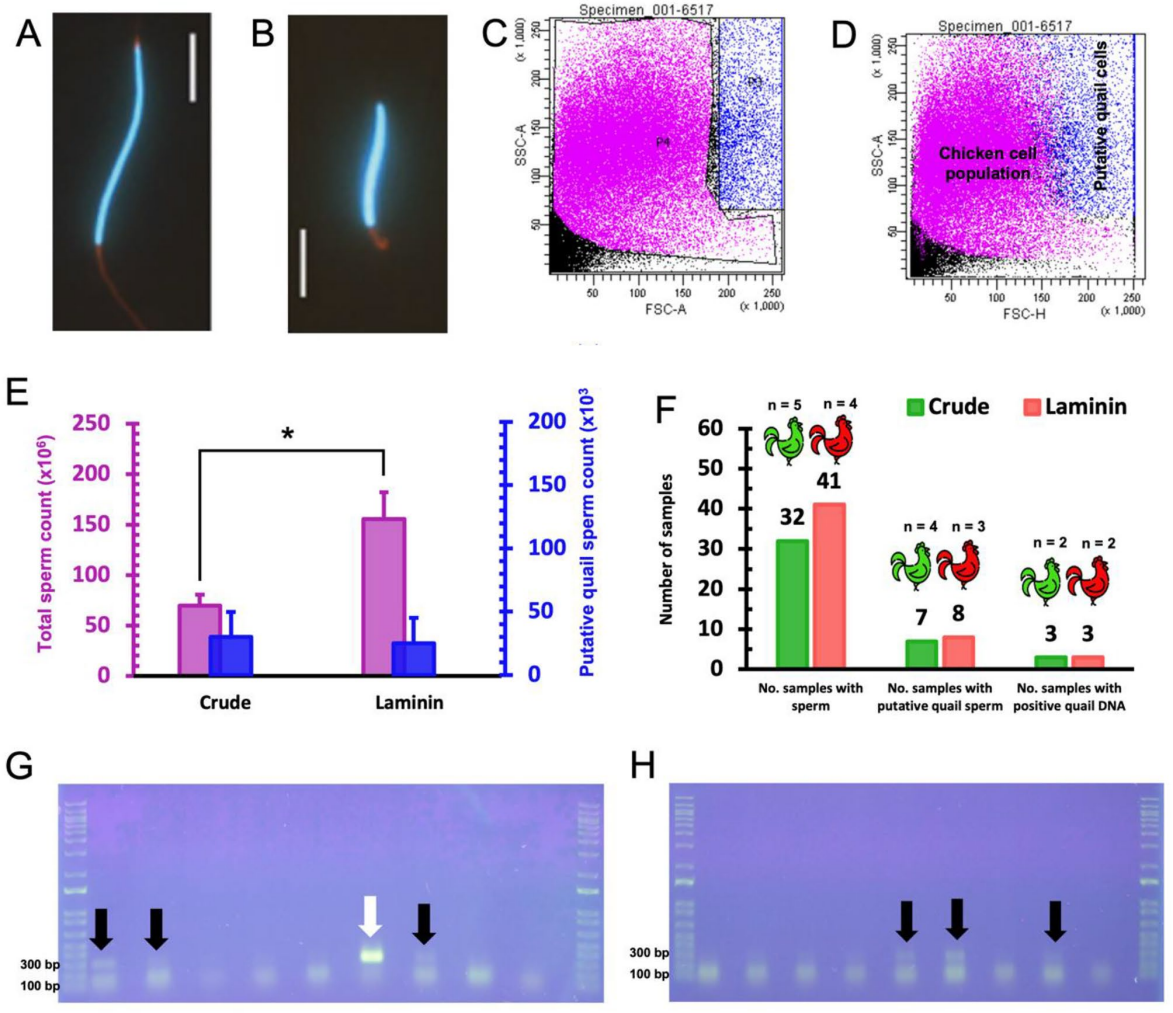
Fluorescence-activated cell sorting (FACS) and PCR analysis of chicken recipient ejaculates. (**A**) Quail sperm morphology stained with Hoechst 33,342. (**B**) Chicken sperm morphology stained with Hoechst 33,342 stain. (**C**,**D**) Subpopulations of chicken and putative quail spermatozoa found in ejaculates of some recipients transplanted at 9–10 weeks of age (for further information regarding flow cytometry gating strategy, please refer to Supplementary Material Fig. S2). (**E**) Total and putative quail sperm counts of ejaculates from chicken recipients considered positive during FACS (asterisks indicate statistically significant differences in sperm counts from recipients injected with crude testicular cells and spermatogonia-enriched fractions—P < 0.05). (**F**) Total number of ejaculates, number of ejaculates with putative quail spermatozoa (FACS), and number of ejaculates positive for quail DNA (PCR) obtained from recipients injected with crude testicular cells and spermatogonia-enriched fractions. (**G**,**H**) Detection of quail ovalbumin DNA by PCR from spermatozoa collected in the transplanted chicken ejaculate (for further information regarding PCR specificity and sensitivity, please refer to Supplementary Material Fig. S3). The positive control of PCR is detached by white arrow and positive samples are detached by black arrows.

Five months after transplantation, quail cells were detected by immunohistochemistry in 21.4% (3/14) of the recipients treated with 20 mg/kg of busulfan. In most cases, quail cell colonies were located near the basement membrane without any signs of spermatogenic differentiation, whereas tubules with spermatogenic activity were only found among individuals transplanted with spermatogonia-enriched cell fractions (Fig. 7). Moreover, sperm sorting by flow cytometry (FACS) enabled the identification and separation of putative quail cells in seminal samples from 4 recipients, which we later confirmed to contain quail DNA by PCR (Fig. 8). Spermatozoa with quail DNA were detected in 8.2% of the samples collected throughout 13 weeks, of which an average of 15,000 events per sample were sorted (representing approximately 2.8% of the total number of cells counted per positive sample). Details about gating strategy in FACS and detection of quail genomic DNA are provided in Supplementary Figs. S2 and S3.

## Discussion

Most literature on germline chimera production in birds derives from research with PGCs, and therefore few studies have addressed the preparation of immature or adult recipients for SSC transplantation^1,21,23,30^. This in turn hinders the full applicability of SSC technology in avian species, since the use of practical and safe protocols for the depletion of endogenous germ cells in recipients represents a critical prerequisite to improve efficiency of this technique^11,24^. However, despite the existing knowledge, no information is available regarding the preparation of neonates in birds, an approach that in mammals has proved to increase SSC niche availability and accessibility^24,26^. Herein, we demonstrated that fractionated chemotherapy (FC) constitutes a feasible strategy to deplete endogenous germ cells in newly hatched chicks, minimizing overall side effects and mortality. All losses registered in this study occurred due to accidental injections into the gut during the first week of treatment, which led to deaths even in the vehicle group. These findings are in agreement with previous work with pubertal-age roosters, in which individuals exhibited better tolerance to multiple doses of busulfan than to single-dose applications in terms of survival rate^23^.

Although slower weight gain during FC was expected, chicks only displayed lower body weights after 4 weeks of age. Effects of busulfan on growth performance were earlier described by Honaramooz et al.^29^, who observed that piglets from busulfan-treated pregnant sows were smaller and had reduced weight gain during the first 6 weeks when compared to their untreated counterparts. However, immature roosters presented slighter declines in body weight when equal amounts of busulfan were applied in two doses instead of single injections (i.e. 20 + 20 mg/kg rather than 40 mg/kg), implying that fractionated regimens are physiologically less stressful for recipients^23^. Furthermore, our protocol did not seem to seriously disturb erythropoiesis as moderate decreases in hematocrit were only perceived in VE and BU10 groups, outcome considered by us promising in view of the deaths by severe anemia formerly documented in mice, pigs and roosters following large doses of busulfan^22,29,31^. Side effects linked to the vehicle (DMSO) have been also suggested in piglets and roosters^23,29^. However, it remains unclear whether the decrease of hematocrit levels shown here can be interpreted like a disturb erythropoietic caused by DMF or busulfan toxicity once that layer hens can exhibit a reference interval of their hematocrit levels (i.e. 18 to 31%)^32^ totally consistent to reported by us. Another element that possibly mitigated busulfan effects on general health was the preparation of hatchlings rather than adult roosters as recipients. This concept was based on previous data wherein pediatric maximum doses for 16 chemotherapeutic agents (including busulfan) exceeded adult maximum doses due to children’s faster metabolism and clearance^28^. Hence, it seems reasonable to assume that similar mechanisms may exist in newly hatched chicks conferring them greater tolerance to chemotherapy protocols.

Early exposure to busulfan also induced a decline in testosterone levels, but unlike the highest dose, injections at concentrations of 10 and 20 mg/kg did not result in permanent suppression with cockerels restoring steroidogenesis at 15 and 25 weeks of age, respectively. Decreases in testosterone after busulfan treatment have been previously described in fetal and prepubertal rats, but not in adult mice, variance that seems to be related to age at treatment and/or species-specific tolerance of Leydig cells to busulfan^33–36^. Reductions in testosterone output are presumably caused by numerical depletion of Leydig cells which affect directly and indirectly spermatogenesis^34–37^. Consistent with this knowledge, sperm production was initially impaired in roosters treated with 10 and 20 mg/kg, but then it was partially recovered alongside the restoration of the steroidogenic activity. Nevertheless, similarly to testosterone levels, spermatogenesis in individuals injected with 40 mg/kg was not re-established by the end of the study. Thus, we believe that the highest dose led to a more persistent spermatogenesis damage not only by its direct impact on germ cells, but also by its impact on somatic cells (particularly on Leydig cells). These side effects may represent a serious problem in studies involving germ cell transplantation, since SSC homing and expansion depend greatly upon somatic support cells (i.e. Sertoli, Leydig and myoid cells)^26^. This, together with previous data defining 30 mg/kg as an optimal dose to prepare recipient mice^31,38–40^, suggests that fractionated doses of 20 mg/kg or slightly higher (e.g. 30 mg/kg) may be more adequate to ensure germ cell depletion without harming somatic support cell populations in chicken testes.

Dose-dependent decreases in GSI as well as in seminiferous tubule diameter and score were likewise noticed following our chemotherapy regimens. These responses were not surprising given that testes and seminiferous tubules lacking spermatogenesis are known to reduce largely in size^1,19,23,29,34–36,39–41^. Notwithstanding, the highest dose was clearly detrimental to seminiferous epithelium at 6 weeks of age, as histology revealed empty tubules or tubules comprising dead cells in most part of the parenchyma. Concurrently, multiple injections of 20 mg/kg appeared to provide the desired effect on germ cells without damaging testes somatic cells, whereas 10 mg/kg was apparently not sufficient to deplete germ cells. Subsequent analyses at 30 weeks of age confirmed the long-term deleterious impact of busulfan at 40 mg/kg on testes weight and morphology, given the virtual absence of haploid cells in seminiferous tubules. By contrast, roosters treated with 10 and 20 mg/kg re-established germinal epithelium at different degrees, indicating that more germ cells were able to survive the toxic effects of busulfan at the lowest dose. Once again, these findings supported the idea that fractionated doses of busulfan between 20 and 40 mg/kg are perhaps the most suitable chemotherapy protocol for the preparation of chick recipients.

Our IHC analysis revealed that busulfan at 10 mg/kg greatly reduced testicular cells expressing NANOG, whereas NANOG expression was detected in hardly any and no cells at doses of 20 and 40 mg/kg, respectively. In mammals, NANOG is a homeodomain transcription factor required for maintaining pluripotency in embryonic stem cells (ESCs), and whose postnatal expression in testes of several species implies its involvement in the activity of undifferentiated germ cells^42–46^. In chickens, NANOG is expressed in individual cells scattered over the epiblast at HH1 and HH3 stages (allegedly PGCs), and it becomes restricted to germ cells during late embryonic development^47–49^. Therefore, antibodies against NANOG were chosen to examine the efficiency of FC in reducing the presence of endogenous germ cells in chick testes. However, unlike the strong nuclear staining formerly reported in chicken PGCs^47^, NANOG expression in germ cells of 6-week-old chicks was solely detected in the cytoplasm. Similar cytoplasmic immunolocalization of NANOG protein was previously seen in gonocytes and spermatogonia of postnatal testes of mice, pigs, bulls and blackbucks^42,45,46^. According to Goel et al.^42^, this postnatal expression seems to vary as age progresses, and NANOG translocation from the nucleus to the cytoplasm may be related to its eventual degradation.

In parallel, PGC and SSC markers were assessed by RT-PCR in order to ratify our IHC results. Despite the absence of differential expression for *GFRα1*, chicks submitted to doses of 20 and 40 mg/kg showed lower *Nanog* and *c-Myc* expressions suggesting a decline of endogenous germ cells. *Nanog* and *c-Myc* are frequently used as stem cell markers, but their expressions have been also associated with PGCs, gonocytes and proliferating spermatogonia, and seem to progressively decrease as differentiation proceeds^42,43,46,50–55^. Nevertheless, the fact that *GFRα1* expression did not drop after FC is intriguing and differs from previous findings in rhesus macaques, where expression of this marker was absent following busulfan treatment^56^. It may be possible that expression patterns of *GFRα1* vary in an age-dependent manner leading to inaccurate readings. Researches have observed that the expression pattern of *GFRα1* changed during postnatal development in mice^57^, and such phenomenon could explain why we were unable to detect differences among treatments. Moreover, we do not exclude the possibility that *GFRα1* was only expressed by a subpopulation of spermatogonia (possibly SSCs) which was not affected by busulfan. Such assumption is partially supported by earlier studies with neonatal and adult mice that showed the existence of *GFRα1*-*positive* and *GFRα1*-*negative* subsets of undifferentiated spermatogonia^58–60^.

Bearing in mind that busulfan induces a G2 cell cycle block and apoptosis in rapidly proliferating cells^61^, we also became interested in investigating pro- and anti-apoptotic markers in testes after FC. In short, *ch-Bid* expression did not change regardless of dose-dependent increases in *Bcl-2* expression. These results suggest that Bu-induced spermatogenic cell death possibly reached its plateau at the end of the treatment, leaving only a small subpopulation of cells that was resistant to the cytotoxic effects of this alkylating agent. It seems likely that these Bu-resistant cells are in fact SSCs who accounted for the GFRα1 expression detected in treated groups, since both features (*GRFα1* expression and chemotherapy resistance) have been formerly attributed to SSCs^57,62–64^. Besides, we believe that the increasing *Bcl-2* expression observed in treated chicks could be related to some protection mechanism of SSCs against chemotherapy. Survival of SSCs following busulfan treatment was previously described in mice and apparently involves modulation of cell cycle through the inhibition of spermatogonial proliferation (i.e. cell cycle arrest at G0/G1 – Ref.^62^). Up-regulation of *Bcl-2* and other anti-apoptotic genes (*Bcl-xl, Bcl-2L10, Bag3* and *Iap2/Birc3*) have been also documented in Bu-resistant leukemia cell lines, which are capable of evading busulfan mediated G2-arrest and apoptosis^65^. However, the marked rise in *Bcl-2* expression registered at the highest dose (BU40) combined with our observations of widespread tubular degeneration indicate that this protocol may not be ideal to prepare recipients due to its impact on somatic cells and, consequently, SSC niche.

Finally, we tested the capacity of the testes from chicks treated with 20 mg/kg busulfan to support xenogeneic spermatogenesis. Quail testicular cells were chosen because of their traceability in the chicken testis by QCPN antibody and due to morphological differences between quail and chicken spermatozoa^66^. Our FACS analysis showed that some recipients’ ejaculates had distinct sperm populations, a finding later corroborated by the detection of quail genomic DNA. Besides, we were able to observe quail germ cells in histological sections of chicken testes. Although previous studies had already reported the ability of quail cells to colonize the gonads of embryos and adult chickens^19,67^, this is the first time that complete spermatogenesis in the quail-chicken model has been registered. It is plausible to assume that this achievement may be related to the use of young recipients since tubular compartmentalization triggered by Sertoli cells was not yet consolidated at the time of transplantation. This hypothesis is supported by the findings of Shinohara et al.^24^ in mice, where the microenvironment of neonatal testes was 9.4 times more favorable to colonization of donor cells than adult testes. For these authors, the blood-testis barrier (BTB) present in the testes of adult recipients hampers the access of donor cells to the seminiferous tubule basement membrane. Recent studies also suggest that BTB is dispensable for spermatogenesis because infertile mice without BTB become fertile after germ cell depletion^68,69^.

Our data also confirmed that FC in young chicks effectively impaired endogenous spermatogenesis which, in turn, increased the number of vacant stem cell niches. Chemotherapy regimens employed here were physiologically less aggressive for both recipients and donor cells as possible residual effects of busulfan might have been reduced due to higher drug metabolism and clearance. This chemotherapeutic agent is regularly used prior to stem cell transplantation because of its preferential damages on type A spermatogonia^31^. Nevertheless, busulfan can also disrupt Sertoli cells and even reduce BTB protein expression^70^. Because Sertoli cells in recipient chicks might have been damaged by busulfan treatment, we assessed the effects of transplantation with crude testicular fractions in which somatic and germ cells are injected together. In another group, quail cells underwent laminin selection in order to enrich the number of spermatogonia transferred per testis. Interestingly, the number of ejaculates and recipients exhibiting quail DNA was almost the same regardless of the cell fraction transferred. These outcomes imply that the remaining Sertoli cells in recipient testes were sufficient to support germ cell colonization and differentiation, but the enrichment of spermatogonia by laminin plating was insufficient to improve transplantation efficiency.

PCR-confirmed quail spermatozoa were present in less than 9% of the ejaculates collected, and in these ejaculates less than 3% of the total sperm counted was sorted as quail. Such low rates may be the result of the reduced number of viable spermatogonia injected into the testes of the recipients, even more so considering the deleterious effects of cryopreservation and enzymatic digestion on donor germ cells^8^. Added to this is the very low number of spermatogonia recovered after laminin plating (about 3 × 10^5^ cells per plate), which represents only 3,1% of the cells in the crude fraction^19^. These constraints substantiate the need for advances in avian SSCs culture systems in order to optimize the efficiency of interspecific transplants for rare and endangered species. Unfortunately, although in vitro culture is often used to expand PGCs^9,71,72^, this approach with avian SSCs is not widespread and germline transmission still shows a great discrepancy even among allogeneic transplants in quails^73^.

In conclusion, we have shown that FC using busulfan at a dose of 20 mg/kg is a simple, inexpensive, and safe method for the preparation of chick recipients to be used in SSC transplantation. However, although successful, a bottleneck for the efficient application of this technique in wild bird conservation is the available number of donor SSCs. Therefore, future efforts are required to improve the culture of donor SSCs with the aim of increasing the cell colonization of recipient testes and, consequently, germline transmission.

## Material and methods

### Ethical statement

All animal experiments were performed in accordance with relevant guidelines and regulations and received approval from the University of Sao Paulo Institutional Animal Care and Use Committee (CEUAVET/FMVZ-USP) by protocol no. 2678/2017. All methods are reported in accordance with ARRIVE guidelines. Fertilized eggs were cultured in humidified rocking incubators at 37.5 °C and chick embryos were sacrificed by decapitation. Six weeks old chicks were euthanized by decapitation. Roosters were euthanized by cervical dislocation.

### Experimental design

In the first experiment, one hundred chicks (Hisex White) were randomly assigned into five experimental groups: control (CON); vehicle injections (VE); and busulfan injections at concentrations of 10, 20 and 40 mg/kg (BU10, BU20, and BU40 groups, respectively). Excepting controls, all individuals were treated throughout 5 weeks receiving weekly injections of vehicle (VE) or busulfan (Bu groups). During treatment, effect of busulfan on chicks was assessed weekly by survival and performance parameters. Since our mid-term goal is to perform SSC transplantation in young birds to improve homing and expansion of donor cells, ten recipients from each group were euthanized following chemotherapy (6 weeks of age) for blood and testes collection with the purpose of evaluating their treatment response before cessation of Sertoli cell proliferation (i.e. before 8 weeks of age^74^). Blood processing consisted of hematocrit tests, whereas testes were weighted and sent to histological examination, immunohistochemistry and RT-qPCR. The remaining recipients (ten birds per group) were kept until 30 weeks of age for measurement of plasma testosterone every 5 weeks and for weekly estimation of sperm production. Later on, these individuals were euthanized and testes were weighed and processed for histological analysis.

In the second experiment, fourteen chicks were depleted with busulfan injections (20 mg/kg) according to the same methodology employed in Experiment 1. After that, young chicks (between 9–10 weeks of age) were divided in two groups: (1) individuals injected with thawed quail testicular cells after enrichment of spermatogonia by laminin plating^19^ (n = 7); and (2) individuals injected with thawed quail testicular cells without any previous selection (i.e. crude testicular cells). These recipients were kept for semen collection (18–30 weeks of age) and then euthanized for processing of their testicles. Ejaculates and testes from these recipients were investigated for the presence of quail spermatozoa (FACS and PCR) e testicular cells (IHC and PCR), respectively.

### Recipient preparation by busulfan

Busulfan (1,4-butanediol dimethanesulfonate, Bu) and DMF (N, N-dimethylformamide) were obtained from Sigma-Aldrich (St. Louis, MO). Busulfan was firstly dissolved in DMF and then, immediately before administration, an equal amount of distilled water (40 °C) was added to reach the desired concentration. Furthermore, weekly average weights from each experimental group were used to adjust concentrations of busulfan solutions to be injected. Both busulfan and vehicle injections were administered intraperitoneally (volume ranging from 0.3 to 0.5 mL) using tuberculin syringes (Luer slip 1 mL, 0.38 × 13 mm, BD Plastipak) to 40 °C.

### Health monitoring

To evaluate the influence of fractionated doses of busulfan on health status, survival, body weight (BW) and feed intake (FI) were weekly measured throughout the treatment (weeks 1 to 5). Because animals from the same experimental group were housed together, feed intake was calculated as the difference between the feed offered and the existing surplus at the end of the week, divided by the number of chicks per cage. Additionally, because busulfan impairs hematopoietic cell lines^11^ blood samples were collected prior to euthanasia to carry out hematocrit tests.

### Testosterone levels

From 10 to 30 weeks of age, blood samples were collected every 5 weeks, transferred to heparinized polystyrene microtubes (approximately 20 IU sodium heparin/mL), and kept in icebox before separation of plasma (4 °C). At the laboratory, blood samples were centrifuged (4 °C, 3000 g, 10 min) and plasma was stored at − 20 °C until assayed. Enzyme immunoassay protocols (EIA) were performed following Brown et al.^75^ and Pereira et al^76^, and antiserum for testosterone detection was supplied by C. Munro (University of California, CA, USA). To assess the physiological relevance of immunoreactive testosterone, we used data on plasma samples from adult chicken males and females (138.50 ± 13.02 and 32.28 ± 8.49 ng/mL, respectively – P < 0.05). Intra and inter-assay coefficients of variation for two internal controls (20 and 80% binding) were 9.8% and 15.2%, respectively. Assay sensitivity (calculated at 90% binding) was 0.03 ng/mL.

### Semen analysis

From 18 to 30 weeks of age, semen was collected from roosters once weekly from using the dorso-abdominal massage method^77^. Sperm concentration of clean ejaculates (i.e. without feces or urine contamination) was immediately determined by spectrophotometry (Accuread rooster and turkey photometer, IMV, France), and final concentration was achieved by diluting each sample with Lake medium. After dilution, sperm motility was assessed using a Hamilton Thorne Version 12.3 K IVOS (Hamilton Thorne Biosciences, Beverly, MA)^78,79^. Total sperm count was calculated by multiplying the sperm concentration by the volume of the ejaculate.

### Tissue processing

Immediately after euthanasia, testes were collected, weighted and prepared for histological, immunofluorescence and molecular analysis. For light microscopy, testes were fixed in 4% paraformaldehyde (4 °C, 12 h), embedded in paraffin, and cut into 4–5 μm sections for hematoxylin–eosin staining. For immunohistochemistry, unfixed testes were washed three times in PBS, immersed in OCT compound (Sakura Finetek, Torrance, CA), quickly frozen in liquid nitrogen, and stored at − 80 °C until processing. Then, cryostat sections of 6 µm thickness were mounted on silane-coated slides (StarFrost, Waldemar Knittel, Germany) and stored at − 20 °C until immunofluorescence staining. For RNA extraction, testis was washed three times in PBS, sliced into small pieces and submerged in RNA holder (BioAgency, Brazil) for 48 h before freezing storage. Before one month of storage, RNA extraction was performed using TRIzol reagent (TRIzol Life Technologies, USA) according to the manufacturer’s instructions. RNA concentration (ng/μl) was determined with ND-1000 Spectrophotometer at 260 nm (NanoDrop®, Thermo Scientific). Only pure RNAs (260/ 280 nm ratio around 2.0) were employed for cDNA synthesis.

### Histological analysis

Stained sections were analyzed in bright field microscopy to measure tubular diameter (at least 50 seminiferous tubules per section – Microscope Software Zen 2 core, ZEISS, Germany) and assess histological changes in testicular parenchyma. Changes in tubules were classified into four scores (Score I: highest damage to Score IV: full normality). Score I: complete absence of Sertoli and germ cells inside the tubules; Score II: presence of Sertoli cells only (containing or not abnormalities – e.g., vacuolar degeneration, blebbing, etc.); Score III: presence of normal Sertoli cells and abnormal germ cells; and Score IV: presence of Sertoli and germ cells without morphological alterations.

### Immunofluorescence staining

Slides were removed from − 20 °C and subsequently fixed in cold 4% paraformaldehyde (PFA) for 10 min. Sections were then rinsed three times in 0.5% Tween PBS (TTBS), and incubated with antiserum against chicken Nanog (cNanog; 1:500^47^) for 2 h at 37 °C. After being rinsed with TTBS three times, the sections were incubated with goat anti-rabbit Alexa Fluor 488 (1:500; Invitrogen, Carlsbad, CA, USA) for 1 h at 37 °C. Forthwith upon incubation with secondary antibody, sections were rinsed three with TTBS, stained with DAPI for 5 min (1:1000; DAPI solution 1 mg/ mL, Invitrogen), and mounted with Vectashiled Mounting Medium (Vector Laboratories, Burlingame, CA). Images were obtained using an epifluorescence microscope (Olympus BX-60) with a combination of excitation and emission filters at 488/650 nm, equipped with a Zeiss AxioCam HRc. As positive controls, embryos from freshly laid eggs (EGK stage X) were collected, washed in Pannett Compton solution and fixed in Sucrose/PFA solution (20% sucrose w/v in PFA 4%) overnight at 4 ºC. Fixed embryos were prepared, cryosectioned and stained using the same protocol described above. In negative controls, primary antiserum was omitted.

### Molecular analysis

To investigate decreases of endogenous germ cells after busulfan treatment, expression of pluripotency, primordial germ cell (PGC) and spermatogonial stem cell (SSC) markers were examined by quantitative reverse transcription PCR (RT-qPCR) with cDNA samples prepared from chicken testes. In parallel, pro- and anti-apoptotic markers were assessed in order to monitor cellular death within testes parenchyma. The target genes were *ch-c-Myc, ch-Nanog, ch-GFR*α1, *ch-Bid* and *ch-Bcl-2,* whereas the *ch-GAPDH* was adopted as reference gene. cDNA was obtained using RNA samples (1 μg per reaction) *and SuperScript® III Reverse Transcriptase* (Invitrogen, EUA) as recommended by the manufacturer. Standardization of primers showed that selected oligonucleotide sequences were efficient for amplification by RT-qPCR. The primers used for the transcript of interest were designed by the computer program Primer Express® Software version 3.0 (Applied Biosystems) or taken from literature (Supplementary material, Table S1). The reagent Fast SYBR® Green Dye (Life Technologies) and ViiA7 Real Time PCR System (Life Technologies) were used for the quantification of the product under the following conditions: 95 °C for 20 s, 40 rounds of 95 °C for 1 s and 60 °C for 20 s. A dissociation cycle was performed after each run to check for nonspecific amplification or contamination. The analysis was carried out as cited in Lobba et al.^80^.

### Isolation and cryopreservation of quail testicular cells

Testis from sexually mature (32-week-old) Japanese quail (*Coturnix japonica*) were retrieved and dissected, and testicular cells were enzymatically isolated and cryopreserved as described by Guan et al.^81^. The cryopreservation medium was based on DMEM supplemented with 20% of fetal bovine serum, 8% DMSO, and 1% Penicillin/Streptomycin.

### Spermatogonia selection by laminin

After thawing and removing the cryopreservation medium, there were selections of spermatogonia by laminin as described by Guan et al.^81^. Briefly, cell culture dishes were coated with 3 mL of laminin solution (20 mg/mL, overnight, 37 °C, 5% CO_2_). On the next day, laminin-coated dishes were washed with PBS and incubated for 1 h with bovine serum albumin (BSA) 0.5 mg/mL. Subsequently, dishes were washed with PBS, and 2 mL of testicular cell suspension (10 × 10^6^ cells) was added and incubated for 15 min (37 °C, 5% CO_2_). Unattached cells were removed by washing with PBS (0.5 mL per 3 times) and discarded. Attached cells were removed (with trypsin/EDTA), washed thrice, and resuspended in 2 mL of DMEM supplemented with 10% of FBS.

### Interspecific transplantation

Prior to cell transplantation, recipients were depleted with 20 mg/kg of busulfan in accordance with the procedure written above. For the transplantation, recipients were anesthetized with isoflurane (3–5% for induction and 1.5–2.5 for maintenance) under 100% O_2_. The access of testis was performed through a small incision (1 cm) on the flank between the sartorius and iliotibial muscles which injection were made into tunica albuginea through chromatography syringe (Hamilton®, USA). The group injected with the crude testicular fraction received 50 µL of cell suspension containing a total of 2 × 10^6^ cells per testis, while the group injected with spermatogonial enriched fraction received 50 µL containing a total of 0.3 × 10^6^ cells per testis. Analgesics, anti-inflammatory, and antibiotic (tramadol 0.1 mg/kg, meloxicam 0.3 mg/kg, dipyrone 0.5 mg/kg and enrofloxacin 5 mg/kg, respectively) were given during 5 days after surgery.

### Immunodetection of quail cells

Tissue was fixed in Serra’s (6:3:1, 100% ethanol: 37% formaldehyde: glacial acetic acid) overnight at 4 °C, dehydrated, paraffin embedded, and cut into 7 µm. Subsequently, slides were deparaffined, incubated twice with 0.1% BSA in PBS (2 min, each), and permeabilized twice with 0.5% Triton X-100 in PBS (5 min, each). For antigenic recovery, slides were heated in microwave oven per 10 min immersed in TRIS–EDTA buffer solution supplemented with 0.05% of Tween 20, while endogen peroxidation was blocked with hydrogen peroxide per 15 min at room temperature in humidify chamber. Nonspecific background was blocked with protein block per 10 min and quail-specific antibody (QCPN concentrate; Developmental Studies Hybridoma Bank, University of Iowa) at concentration of 2–5 µg/mL was carried out overnight 4 °C in humidity chamber. After 2 washes with 0.5% Triton X-100 in PBS (5 min each), the sections were incubated with secondary antibody (horseradish peroxidase-conjugated—HRP) for 30 min and HRP conjugated for 1 h. After, slides were washed again with 0.5% triton X-100 (5 min) and incubated with DAB solution (3,3′ diaminobenzidine) 1 µL/mL for 45 s. Immediately, slides were rinsed with distilled water for 5 min and counterstained with Hematoxylin solution (Sigma-Aldrich, St. Louis, MO) for 5 min. Finally, slides were rinsed in tap water, fixed and mounted with Permount® (Thermo Fisher Scientific, Waltham, MA).

### FACS cytometry quail cell detection

Ejaculates from recipients were collected, immediately diluted in lake medium, and centrifuged at 500 g for 10 min to generate a cell pellet. After centrifugation, the supernatant was discarded and FACs Flow buffer was added. Then, cell suspension was transferred to cytometry tubes and Hoechst 33,342 solution (Thermo Fisher Scientific, Waltham, MA) was added and incubated for 10 min at room temperature. Following incubation, samples were analyzed using FACSAria II flow cytometry (Becton Dickinson (BD) Bioscience, USA) by counting of total events present in the quail isotype gate. The quail isotype gate was previously determined by isolated analysis of quail and chicken sperm, as also by a mixture of spermatozoa of both species in different concentrations (i.e. 25%/75%, 50%/50%, and 75%/25%) (for more details about our gating strategy, please refer to the Supplementary material Fig. S2). All analyses were performed using software BD CELLQUEST.

### Quail sperm DNA detection

FACS positive sperm samples were incubated at 55 °C for 18 h under agitation in 300 µl of DNA lysis buffer (10 mM Tris–HCl pH 8; 5 mM EDTA; 0.5% SDS; 200 mM NaCl), 1.5 ul of Triton X-100, 6 ul of proteinase K (20 mg/mL) and 15 ul of 1 M DTT. After incubation, 300 µl of phenol:chloroform:isoamyl (25:24:1) were added to samples and mixed vigorously. Then, the samples were centrifuged at 17,000*G* for 10 min. Approximately 250 µl of the upper fraction was placed in a new micro tube and added 26 µl of 3 M sodium acetate. Therefore, 550 µl of 100% ethanol was added and gently mixed to samples. The samples were incubated at − 20 °C for 30 min and then, centrifuged at 17,000G for 30 min at 5 °C. The supernatant was discarded and 500 µl of 70% ethanol was added to pellet. The samples were centrifuged at 17,000G for 10 min at 5 °C and then, the supernatant was discarded by inversion. The pellet remained at room temperature until dry completely. The samples were suspended in 30 µl of RNase—DNase free ultrapure water. The total DNA was quantified by Qubit dsDNA HS and Qubit 2.0 Fluorometer (Life Technologies). Primers for quail ovalbumin (Forward Primer: CAGAGGCTGGAGTGGATGCTA, Reverse Primer: TATTACTCTGTGTAAGGGAAGGGTGAAGT) were designed using the online software Primer-BLAST (http://www.ncbi.nlm.nih.gov/tools/primer-blast) based on the sequences of the respective *Coturnix coturnix* transcripts (GenBank No. XP_015709964). The qualitative PCR was carried out to verify the presence of these transcripts in spermatozoa. GoTaq® G2 DNA Polymerase kit (Promega, Madison, USA) was used for PCR in a Mastercycler Nexus Thermal Cycler (Eppendorf AG, Hamburg, Germany) (40 amplification cycles of 95 °C for 15 s, 60 °C for 30 s and 72 °C for 30 s) according to the manufacturer. We used 1% agarose gel electrophoresis to separate the transcripts. Electrophoresis (FB300, Fisher Scientific) was performed for 50 min at 110 V at room temperature. The samples were previously stained with SYBR Safe DNA gel stain (Thermo Scientific, Massachusetts, USA), and then scanned using a MultiDoc-It™ 125 Imaging System LM-20 Transilluminator and Life Science Software Doc-It LS Image Acquisition (UVP, Fisher Scientific).

### Statistical analysis

All data are presented as mean ± standard error. All variables were initially tested to determine variance homogeneity and data normality, and data that did not obey these premises were transformed (log10). Effects of treatments on average body weight, hematocrit, gonadosomatic index (GSI), tubular diameter, tubular score and mRNA expressions were analyzed using ANOVA followed by Dunnet’s test. Other statistical analyses were calculated using a two-tailed Student’s *t* test. All statistical analysis were conducted using SAS System software for Windows 9.3 (SAS Institute Inc., Cary, NC, USA), and a significance level of *P* < *0.05* was used for all tests.

### Supplementary Information

Supplementary Information.

## Data Availability

The datasets generated during the current study are available from the corresponding authors on reasonable request.
