# Supplementary material for: Successful xenotransplantation of testicular cells following fractionated chemotherapy of recipient birds
Source: Sci Rep. 2024 Feb 7;14:3085. doi: 10.1038/s41598-023-45019-0 (PMC10847125; doi:10.1038/s41598-023-45019-0)
Supplement: Supplementary file 1 — Supplementary Information. [file 41598_2023_45019_MOESM1_ESM.docx]

**Supplementary Material**

Title:

**Successful xenotransplantation of testicular cells following fractionated chemotherapy of recipient birds**

Marcel H. Blank^1^, Alison J.T. Kawaoku^2^, Bruno R. Rui^1^, Ana C.O. Carreira^3^, Thais R.S. Hamilton^1^, Marcelo D. Goissis^1^, Ricardo J.G. Pereira^1^

^1^Department of Animal Reproduction, College of Veterinary Medicine and Animal Science, University of São Paulo, Av. Duque de Caxias Norte 255, Pirassununga 13635-900, Brazil.

^2^Hendrix Genetics Brazil, Estrada Municipal Slt-161 Km 08, Salto 13328-400, Brazil.

^3^Cell and Molecular Therapy Center (NUCEL), Medical School, University of Sao Paulo, Rua Pangaré 100, São Paulo 05360-130, Brazil.

**Figure S1.** Validation of NANOG as a chicken germ cell marker. The NANOG antibody has been described by Nakanoh et al.^1^as a chicken embryonic stem cell marker (i-iii). However, NANOG also was detected for us in migratory primordial germ cells (PGCs) and within of the gonads (iv-vi) of the embryos with 5.5 days (stage 28 HH). The red line outlines the embryonic gonads. Scale bar 500μm.

|  | BF | DAPI | NANOG |
| --- | --- | --- | --- |
| stage X-EG&K | 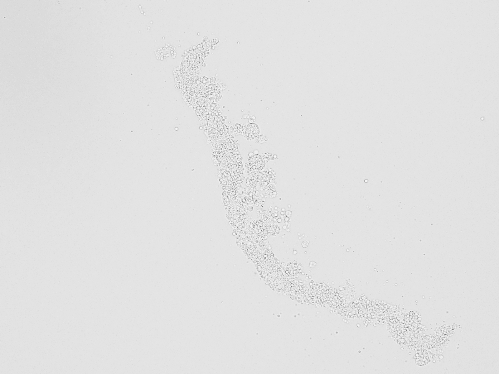  i   | 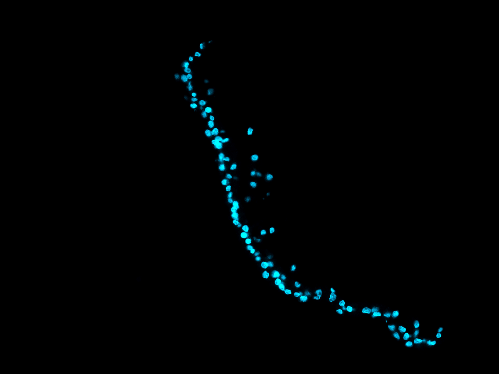  ii | 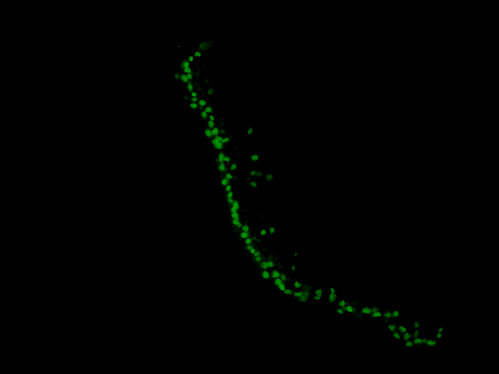  vi  iii |
| stage 28 (HH) | 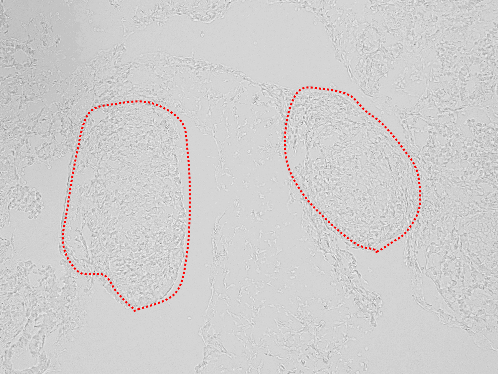  iv   | 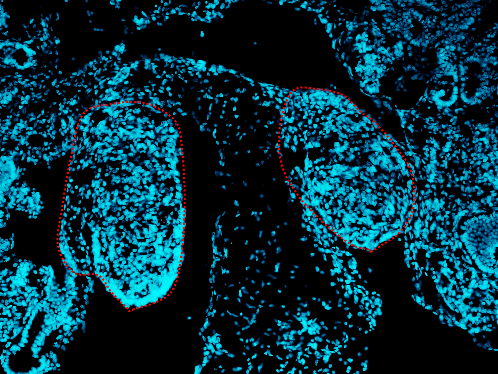  v | 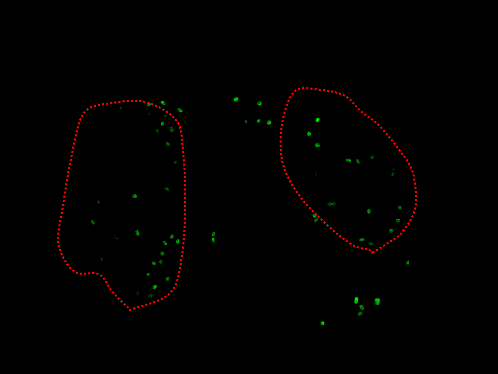 |

**Figure S2.** Gating strategy for the detection of quail spermatozoa in the chicken ejaculates. Prior to flow cytometry, sperm concentration of chicken and quail ejaculates were standardized in 0.1x10^9^ sperm/mL. Then, separately, samples from chicken and quail were stained with Hoechst 33342 solution (0.1 µg/mL) and propidium iodide (5 µg/mL) for 10 minutes at room temperature. Subsequently, a chicken control (A’ and A’’), quail control (B’ and B’’) and 50/50 proportion (v/v) between chicken and quail sperm (C’ and C’’) were made. To access sperm populations in the flow cytometry, dead cells were excluded before through selection of alive cells from the forward/side scatter plot. Due to distinct size and internal complexity of the sperm head, two cell populations were detected whose quail cell population has been defined by P2 gate and chicken cell population by P1 gate. Even though chicken cells also would be found in P2 gate, is expected a greater concentration of quail cells than chicken which subsequently was also validated by PCR through presence or absence of quail DNA in the samples.


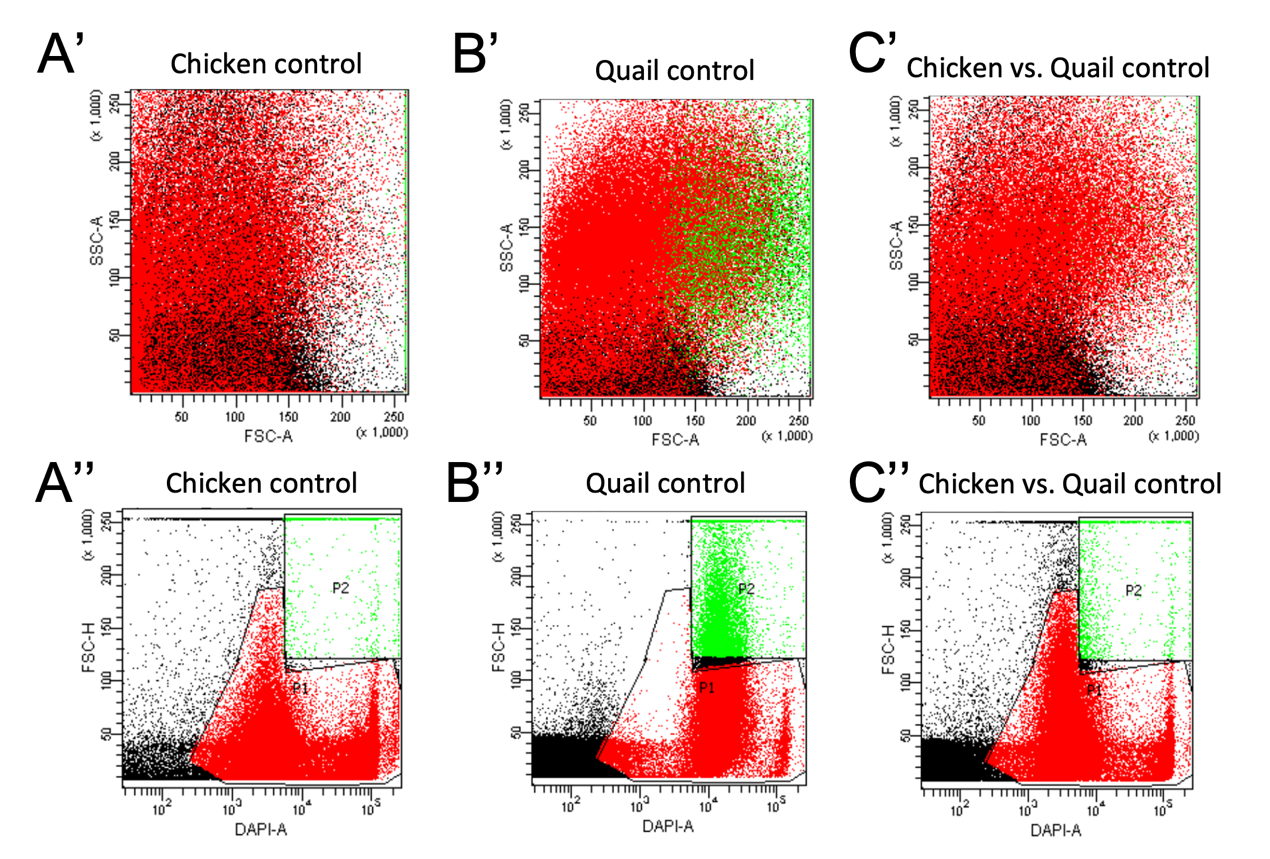


**Figure S3.** Assessing the specificity and sensitivity of PCR reactions for the detection of quail DNA from of pure and mixed ejaculates with chicken spermatozoa after fluorescence-activated cell sorting. (A) PCR specificity test for detection of quail SERPIN14 (ovalbumin) using (1) 0.1 x 10^9^ quail sperm (100 ng DNA per reaction), (2) 1 x 10^5^ quail sperm (10 ng DNA per reaction), (3) 1 x 10^6^ quail and chicken sperm equally mixed (13 ng DNA per reaction), (4) 10 x 10^6^ chicken sperm (100 ng DNA per reaction) and, (5) 0.5 x 10^9^ quail sperm (100 ng DNA per reaction). (B) PCR sensitivity test for detection of quail SERPIN14 in samples with poor concentration of quail sperm. A six-point curve was performed using (1) 1 x 10^5^ quail sperm (10 ng DNA per reaction), (2) 1 x 10^4^ quail sperm (1 ng DNA per reaction), (3) 1 x 10^3^ quail sperm (0.1 ng DNA per reaction), (4) 1 x 10^2^ quail sperm (0.01 ng DNA per reaction), (5) 1 x 10 quail sperm (0.001 ng DNA per reaction), (6) 1 quail sperm (0.0001 ng DNA per reaction) and, (7) negative control using H_2_O. The PCR product was drafted to exhibit a 311bp size.


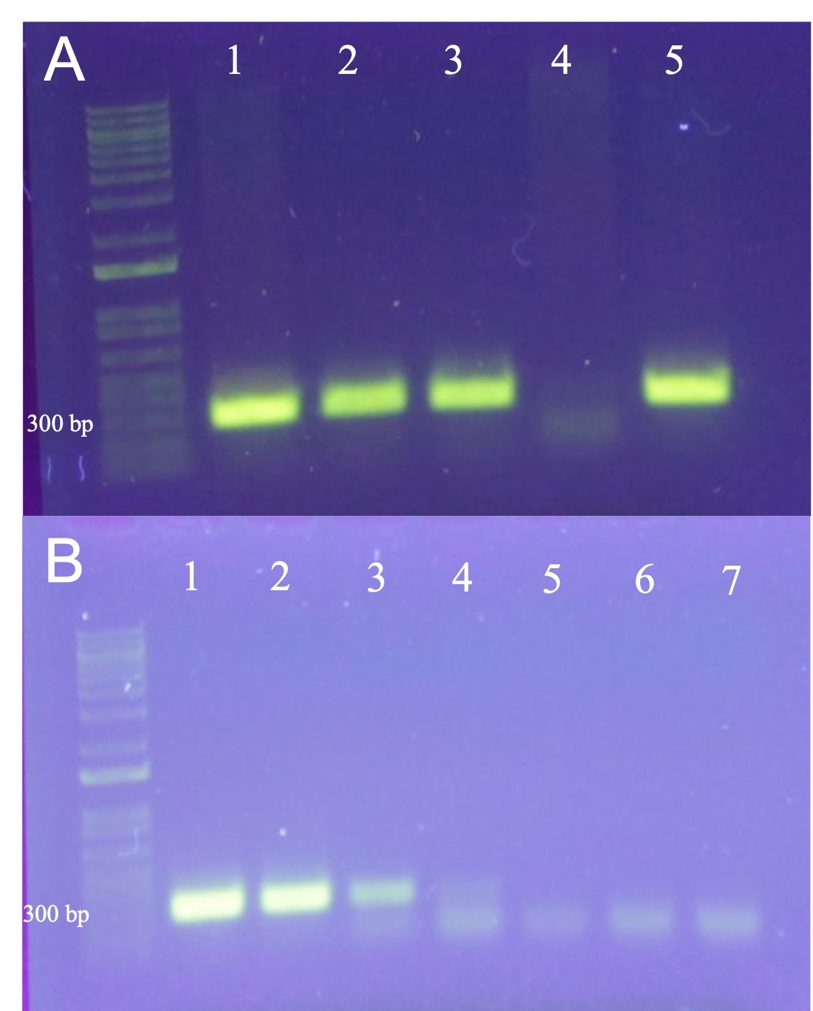


**Table S1.** Standardized primers for quantification of testicular cells expressing marker genes for pluripotency, primordial germ cells, spermatogonial stem cells, apoptosis and anti-apoptosis.

| **Gene** | **Oligonucleotide** | **Sequence (5’ – 3’)** |
| --- | --- | --- |
| ***GAPDH**** (endogenous gene) | Sense primer  Antisense primer | CATCGTGCACCACCAACTG  CGCTGGGATGAT GTTCTGG |
| ***Nanog***** (primordial germ cell marker) | Sense primer  Antisense primer | CAGCAGACCTCTCCTTGACC  TTCCTTGTCCCACTCTCACC |
| ***c-Myc****** (pluripotency marker) | Sense primer | TTCCATCGGTCAGGACACAG  TCATTACGCCTCTGACGCTC |
|  | Antisense primer |  |
| ***GFRα1**** (spermatogonial marker) | Sense primer  Antisense primer | GAGGCGGCAGACTATTGTTC |
|  |  | GGAGGCAGTCAGCGTAGTTC |
| ***Bid*** (apoptosis marker) | Sense primer  Antisense primer | AGCTTGCTGAGATCGGAGAC  GGCACCGTGTTATCTCCTCT |
| ***Bcl2*** (anti-apoptosis marker) | Sense primer  Antisense primer | AGGATGGGATGCCTTTGTGGA  CAGGCTCAGGATGGTCTTCAG |

*Mucksová et al.^2^/ **Cañón et al.^3^; Lavial et al.^4^/ ***Yu et al.^5^

References:

1. Nakanoh, S., Fuse, N., Takahashi, Y. & Agata, K. Verification of chicken nanog as an epiblast marker and identification of chicken pouv as pou5f3 by newly raised antibodies. *Dev. Growth Differ.* **57**, 251–263 (2015).
2. Mucksová, J., Kalina, J., Bakst, M., Yanm H., Brillard, J.P., Benešová, B., et al. Expression of the chicken GDNF family receptor α-1 as a marker of spermatogonial stem cells. *Anim Reprod Sci.* **142**, 75–83 (2013).
3. Cañón, S., Herranz, C., Manzanares, M. Germ cell restricted expression of chick Nanog. *Dev Dyn*. **235**, 2889–94 (2006)
4. Lavial, F., Acloque, H., Bertocchini, F., MacLeod ,D.J., Boast, S., Bachelard, E., et al. The Oct4 homologue PouV and Nanog regulate pluripotency in chicken embryonic stem cells. *Development* **134**, 3549–63 (2007).
5. Yu, M.Y., Lian, S., Han, H.B., Yu, K., Li, G.G., Lian, Z.X., Ning, L. Four recombinant pluripotency transcriptional factors containing a protein transduction domain maintained the in vitro pluripotency of chicken embryonic stem cells. *Sci China Life Sci*. **56**, 40-50 (2013).
